# Associations between Physical Activity and Comorbidities in People with COPD Residing in Spain: A Cross-Sectional Analysis

**DOI:** 10.3390/ijerph17020594

**Published:** 2020-01-16

**Authors:** Sheila Sánchez Castillo, Lee Smith, Arturo Díaz Suárez, Guillermo Felipe López Sánchez

**Affiliations:** 1 Faculty of Sports Sciences, University of Murcia, 30720 Murcia, Spain; ardiaz@um.es; 2 The Cambridge Centre for Sport and Exercise Sciences, Anglia Ruskin University, Cambridge CB5 8DZ, UK

**Keywords:** lung disease, physical exercise, prevalence, adults

## Abstract

There is a high prevalence of comorbidities among patients with chronic obstructive pulmonary disease (COPD). Comorbidities are likely common in patients with any COPD degree and are associated with increased mortality. The aim of this study was to determine the prevalence of thirty-one different COPD comorbidities and to evaluate the association between physical activity (PA) levels in people with COPD residing in Spain. Cross-sectional data from the Spanish National Health Survey 2017 were analysed. A total of 601 adults (52.2% females) with COPD aged 15 to 69 participated in this study. PA (exposure) was measured with the International Physical Activity Questionnaire (IPAQ) short form and comorbidities (outcomes) were self-reported in response to the question “Have you ever been diagnosed with…?” Multivariable logistic regression, in three different models, was used to assess this association. Results showed a high prevalence of comorbidities (94%), these being chronic lumbar back pain (38.9%), chronic allergy (34.8%), arthrosis (34.1%), chronic cervical back pain (33.3%), asthma (32.9%) and hypertension (32.8%) the most prevalent. Low PA level was significantly associated with urinary incontinence (2.115[1.213–3.689]), chronic constipation (1.970[1.119–3.459]), cataracts (1.840[1.074–3.153]), chronic anxiety (1.508[1.002–2.269]) and chronic lumbar back pain (1.489[1.044–2.125]). Therefore, people with COPD should increase their PA levels in order to reduce their risk of comorbidities and increase their quality of life.

## 1. Introduction

COPD (chronic obstructive pulmonary disease) is an important challenge for public health. Its increasing prevalence, high morbidity and socioeconomic burden are some examples of its importance [1]. Moreover, a large body of literature shows that COPD is associated with a decline in a patient’s quality of life [2]. COPD is the fourth largest cause of global death [3,4] and will become the third by 2020. In 2012, greater than three million people died because of COPD (6% of all global mortality).

In Spain, the prevalence of COPD in people aged 40 to 80 was found to be 10.2%, being higher in men (15.1%) than in women (5.7%). [5]. This study used the definition of COPD proposed by the GOLD criteria, where the ratio between forced expiratory volume in the first second (FEV1) and forced vital capacity (FVC) is less than 0.70 post-bronchodilator. Considering these findings, it was extrapolated that 2,185,764 people in Spain suffer from COPD [6]. A total of 10% of primary care consultations, 40% of pneumonology consultations and 7% of Spanish annual hospitalizations are due to COPD. Moreover, in people with COPD comorbidities, the most prevalent comorbidities are cardiovascular, metabolic, musculoskeletal and psychological comorbidities [7]. The presence of comorbidities likely drives the high hospitalization rate [8]. In order to reduce the economic burden of COPD, such comorbidities should be prevented. Comorbidities can occur in patients with any degree of COPD and they are associated with increased mortality [9]. The presence of comorbidities between patients with COPD is high. It was found that 80% of COPD patients had at least one comorbidity [10]. However, generally multiple comorbidities coexist. In 2009, Barr et al. found an average of nine comorbidities in COPD patients [11].

Regular and sustained participation in physical activity (PA) aids in the prevention of several chronic diseases, in relation to both primary and secondary prevention. Indeed, secondary prevention is particularly important for those with COPD [12,13].

PA is limited by COPD [14,15], even in early stages [16,17]. This is related to a high risk of hospitalization and readmission [18] and even death [19,20]. However, this limitation in levels of PA in patients with COPD is not only conditioned by respiratory functional impairment. There are other determinants that affect patients’ PA levels like age, dyspnoea, hyperinflation and peripheral muscle weakness [21]. Furthermore, PA level was found to be impaired by the presence of comorbidities independent of the degree of airflow limitation and of the type of comorbidity [10,22].

Nonetheless, participation in regular PA is associated with a better quality of life [23,24,25] and fewer morbidities in people with COPD [18,26]. In a prospective Spanish cohort study carried out in Barcelona, people with COPD who walked for at least one hour per day had a lower risk of hospitalization by COPD exacerbation [24]. Recently, another prospective observational study showed improvements in both the number of exacerbations and the quality of life in COPD patients participating in a walking program [23]. Despite this, a scarce body of literature on people with COPD shows a tendency towards a sedentary lifestyle [27,28].

Thus, it could be hypothesized that people with COPD who practise less PA have an increased risk of developing comorbidities than people with COPD who practise more PA. Therefore, the aim of this study was to determine the prevalence of COPD comorbidities and to evaluate the association between PA level and the risk of comorbidities in people with COPD residing in Spain in order to inform the promotion of PA in the treatment and prevention of COPD.

## 2. Materials and Methods

### 2.1. Study Design

The present study is of a cross-sectional design written following the STROBE checklist (https://www.strobe-statement.org) [29].

### 2.2. The Survey

Data from the Spanish National Health Survey 2017 were analysed. This survey was undertaken in Spain between October 2016 and October 2017. Details of the survey method have been published elsewhere [30]. In brief, for the data collection, a stratified three-stage sampling was used in which the census sections were first considered, then the family dwellings and then an adult (15 years or more) was selected within each dwelling. The dwellings were selected by systematic sampling and to select the person who completed the Adult Questionnaire, the random Kish method was used. The method of data collection used was computer-assisted personal interviewing (CAPI), conducted in the homes of the selected participants. The interviewers, previously trained, completed the questionnaires with the information provided by the participants. All participants signed an informed consent form before responding to survey questions. The present study was conducted in accordance with the Declaration of Helsinki of 1961 (revised in Tokyo in 1989 and in Edinburgh in 2000).

### 2.3. Participants

A total of 601 adults with COPD residing in Spain (314 women and 287 men) were included in the present analyses. To be included adults aged between 15 and 69 years needed to provide an affirmative response to the question “Have you ever been diagnosed with COPD?”. Those adults older than 69 years were not considered, since they did not complete the International Physical Activity Questionnaire (IPAQ) short form. This instrument was primarily designed for population surveillance of PA among adults and it has been developed and tested for use in adults (age range of 15–69 years) and until further development and testing is undertaken the use of IPAQ with older and younger age groups is not recommended [31].

### 2.4. Physical Activity (Exposure)

IPAQ short form was used to measure PA. Total PA MET·min/week were calculated through the following formula: Sum of Walking + Moderate + Vigorous MET·min/week scores. Participants were divided in two categories according to the guidelines for data processing and analysis of the IPAQ [31]: (1) Fewer than 600 MET·min/week and (2) at least 600 MET·min/week, equivalent to meeting current PA recommendations. IPAQ has been validated in adult populations from different countries showing acceptable validity (ρ = 0.30, 95% CI: 0.23–0.36) and reliability (Spearman’s ρ = 0.81, 95% CI: 0.79–0.82) [32]. IPAQ short form has also been validated in the Spanish adult population showing a moderate correlation for total amount of PA (r = 0.277; *p* < 0.05) with 75% of sensibility and specificity (k = 0.33) [33].

### 2.5. Comorbidities (Outcomes)

A total of 31 comorbidities available in the Spanish National Survey 2017 were considered. Those who answered affirmatively to the question “have you ever been diagnosed with… (each comorbidity studied)?” were considered to have that comorbidity. Moreover, the vast majority of the studied diseases were considered in previous literature as COPD comorbidities [7,10,34,35].

Comorbidities were classified in 13 different groups according to the international classification of the diseases (ICD): Cardiovascular diseases, musculoskeletal disorders, immunological disease, respiratory disease, digestive problems, urogenital diseases, eye problems, dermatological problems, mental health problems, neurological disorder, neoplasias, endocrinal and metabolic diseases and permanent injuries [36].

### 2.6. Covariates

The selection of the control variables was based on past literature [37,38]. Sociodemographic variables included age, sex, education and marital status. Education level was based on the highest educational level achieved and was categorized as ≤primary, secondary and ≥tertiary. Marital status was categorised as married or not married (single/widow/divorced/separated). The following variables were also included as covariates: Smoking habits, alcohol consumption and body mass index (BMI). Smoking habit was classified into three groups: Never, former and current smoker. Alcohol consumption was treated as a dichotomous variable: Yes or no. Height and weight were self-reported and used to calculate BMI as weight in kilograms divided by height in meters squared. Obesity was defined as BMI ≥ 30 kg/m^2^. The presence of other comorbidities was also treated as a dichotomous variable: Yes (if they had one or more comorbidities) and no (if they had no comorbidities). For medication intake twenty-three different medicaments were considered: Flu/cold medication, pain medication, fever medication, vitamins/minerals/tonics, laxatives, antibiotics, sedatives, allergy medication, diarrhoea medication, rheumatism medication, heart medication, blood pressure medication, digestive problems medication, antidepressants, contraceptive pill, menopausal hormones, slimming medicines, cholesterol medication, diabetes medication, thyroid medication, naturist products and other. This variable was also treated as a dichotomous variable: Yes (if they had taken at least one of this medicines in the last two weeks) and no (if they had not taken any medicine in the last two weeks).

### 2.7. Statistical Analysis

Descriptive statistics were used to describe sample characteristics. Frequency and percentage were used for categorical variables (sex, education level, marital status, smoking habits, alcohol consumption, obesity, presence of comorbidities and PA) and mean and standard deviation (SD) were used for continuous variables (age). To describe the prevalence of each comorbidity and group of comorbidities descriptive statistics were used. Significant differences in sample characteristics between groups were examined using chi squared tests. 

Multivariable logistic regression analyses were used to assess the association between PA (exposure) and comorbidities (outcomes). The analyses were carried out in three different models. The first model was not adjusted; the second model was adjusted for age, sex, education, marital status, smoking, alcohol consumption and obesity; and the third model was adjusted for the same variables as model two and also for the variables “presence of other comorbidities” and “medication intake”. COPD comorbidities that were significantly associated with PA in model 1 were also analysed in model 2 and COPD comorbidities that were significantly associated with PA in model 2 were also analysed in model 3. All variables were included in the models as categorical variables with the exception of age, which was included as a continuous variable. There were no missing data. Results from the logistic regression analyses are presented as odds ratios (OR) with 95% confidence intervals (CI).

Statistical significance was set at *p* < 0.05. Analyses were carried out with the Statistical Package for Social Sciences (SPSS version 23, IBM, Armonk, New York, USA).

## 3. Results

The sample consisted of 601 adults with COPD residing in Spain. The age range of the sample was 15–69 years, with an average (SD) of 52.8 (14.1) years. A total of 52.2% were women and 47.8% were men. The prevalence of people doing less than 600 MET·min/week was 37.1%. A total of 94% of the people with COPD had comorbidities. An average of six comorbidities at the same time was found. Sample characteristics are shown in Table 1.

The differences between groups were significant for education, marital status, alcohol, obesity, presence of comorbidities, medication intake and PA.

Overall, the prevalence of comorbidities among those with COPD are shown in Table 2. Chronic lumbar pain, chronic allergy, arthrosis, chronic cervical pain, asthma and hypertension were the comorbidities with higher incidence, all of them with more than 30%. Considering ICD classification 56.2% of COPD patients suffered from musculoskeletal disorders, followed by cardiovascular diseases (48.8%) and endocrinal and metabolic diseases (40.8%).

Associations between PA and the studied COPD comorbidities (Table 3) show that, when models were adjusted for sex, age, education level, marital status, smoking, alcohol consumption and obesity, less than 600 MET min/week of PA was associated with significantly higher odds for urinary incontinence (OR = 2.179; 95% CI = 1.251–3.796), chronic constipation (OR: 2.023; 95% CI = 1.150–3.558), cataracts (OR = 1.918; 95%CI = 1.122–3.279) and osteoporosis (OR = 1.713; 95% IC = 0.958–3.064). Chronic lumbar pain, depression and chronic anxiety showed significant odds too. However, when models were adjusted considering also the presence of comorbidities and the medication intake, PA was significantly associated only with urinary incontinence, chronic constipation, cataracts, chronic lumbar pain and chronic anxiety. When models were not adjusted, myocardial infarction had one of the highest odds, but it was not significant (OR = 2.171; 95% CI = 0.844–5.586).

## 4. Discussion

To our knowledge, this is the first Spanish representative study investigating associations between levels of PA and the presence of thirty-one different COPD comorbidities. The results of this study confirm that low levels of PA are associated with higher risk of comorbidities, specifically, urinary incontinence, chronic constipation, cataracts, chronic anxiety and CBP lumbar.

The prevalence of comorbidities in the present study was high, with CBP lumbar (38.9%), chronic allergy (34.8%), arthrosis (34.1%), CBP cervical (33.3%), asthma (32.9%) and hypertension (32.8%) having the highest prevalence. In other countries, hypertension was found to be the most prevalent comorbidity at 48% and 55%, in Swiss and New York COPD patients, respectively [10,11]. Another study carried out in Hungarian COPD patients showed higher prevalence of hypertension (57%), coronary artery disease (21%) and diabetes (12%) [39]. The higher prevalence of the comorbidity hypertension in Switzerland, Hungary and America compared to Spain is likely owing to the antihypertensive effect of the Mediterranean diet that is consumed in Spain. The high prevalence of comorbidities in Spanish COPD patients observed in the present study is worrisome when considering the profound implications this will have on patients’ quality of life, health care management and health care expenditure. Interventions are urgently required to prevent and manage/reduce comorbidities in this population.

To the best of the authors’ knowledge only one other study has investigated associations between levels of PA and the presence of comorbidities among those with COPD [40]. This study aimed to evaluate longitudinal associations between PA and risk of seven categories (cardiovascular, neurological, endocrine, musculoskeletal, malignant, infectious and mental disorders) in 409 COPD patients from the Netherlands and Switzerland. The study suggests that those with high PA levels are less likely to develop depression or anxiety is [40]. That concurs with the present study, where it was also found that lower levels of PA are significantly associated with higher risk of depression and chronic anxiety.

Multivariable logistic regression showed that in the present study performing fewer than 600 MET·min/week was associated with 110.6% increased odds of urinary incontinence, 97.2% of chronic constipation, 82.5% of cataracts, 50.8% of chronic anxiety and 48.7% of CBP lumbar. The risk of developing these comorbidities in COPD patients is higher when compared with other studies in healthy populations. A longitudinal study showed that older women performing 6.2 MET·h/week or less was associated with 4% increased risk of urinary incontinence (OR = 1.04 CI 95% = 0.92–1.18) [41]. In a randomized controlled trial follow-up during 12 weeks, it was found that PA improved defecation pattern in middle-aged inactive subjects, reducing Rome criteria for constipation by 37% [42]. In relation to cataracts, a recent study, has shown that performing fewer than 600 MET·min/week of PA was associated with 57.9% increased odds of cataracts in older adults [43]. In a systematic review and meta-analysis, it was found that when cohort studies were considered, people who practiced a medium level of PA had a 10% lower risk of CBP lumbar (*p* = 0.0009). When cross-sectional studies were considered, the association suggested 7% decreased odds, but it was not significant (*p* = 0.68) [44].

The main strengths of this study were the large representative sample of COPD people residing in Spain and the use of a validated, reliable and internationally recognised questionnaire to measure PA. Nevertheless, the results of this study should be considered within its limitations. Spanish adults aged over 70 were not considered, as IPAQ short form is designed for the age range of 15–69 years and this questionnaire only considered PA in the last seven days, so it is not possible to analyse the accumulative effect of PA. Assessments of COPD and comorbidities were self-reported and thus bias was potentially introduced into the analyses. COPD prevalence is higher in men and older people; this may be owing to the accumulative effect of other risk factors to which individuals have been exposed. Tobacco smoking and other types of smoke coming from air pollution or biomass fuel constitute especially important COPD risk factors [1]. Recently, it was shown that outdoor air pollutants, like particulate matter and NO_2_ were associated with a more rapid lung function decline, increasing COPD prevalence [45,46]. However, external air-borne hazard and also lung function exacerbation history, were not measured in the survey and therefore could not be adjusted for. Moreover, it is a cross-sectional analysis, so the direction of the association is not known. Therefore, future longitudinal studies are needed to clarify the direction and evaluate also the accumulative effect of PA, lung function, exacerbation and airborne hazards, for example from the mineral fibre atlas [47].

## 5. Conclusions

In conclusion, nine of ten COPD patients residing in Spain present comorbidities, with CBP lumbar, chronic allergy, arthrosis, CBP cervical, asthma and hypertension being the most prevalent. A lower level of PA was significantly associated with a higher risk of urinary incontinence, chronic constipation, cataracts, chronic anxiety and CBP lumbar. While the interaction of COPD with PA is not simple straight forward behaviour but an interactive process, it is recommendable for people with COPD to increase their PA levels in an attempt to reduce risk of comorbidities and increase quality of life.

## Figures and Tables

**Table 1:** Sample characteristics.

Characteristic	Category	*n*	%	*p*
Sex	Men	287	47.8	0.271
	Women	314	52.2	
Education	≤Primary	197	32.8	0.006 *
	Secondary	170	28.3	
	≥Tertiary	234	38.9	
Marital Status	Married	402	66.9	<0.001 *
	Not Married	199	33.1	
Smoking	Current	202	33.6	0.979
	Former	201	33.4	
	Never	198	32.9	
Alcohol	Yes	395	65.7	<0.001 *
	No	206	34.3	
Obesity	No	433	72.0	<0.001 *
	Yes (≥30)	168	28.0	
Comorbidities	Yes	565	94.0	<0.001 *
	No	36	6.0	
Medication	Yes	527	87.7	<0.001 *
	No	74	12.3	
PA	<600 MET·min/week	223	37.1	<0.001 *
	≥600 MET·min/week	378	62.9	

*n*: Sample size; %: Percentage; *p*-values were based on chi-squared tests. * *p* < 0.05.

**Table 2:** Prevalence of comorbidities in people with COPD.

	Comorbidities	*n*	%	%
Cardiovascular diseases	Hypertension	197	32.8	48.8
	Myocardial infarction	18	3.0	
	Angina, Coronary HD	26	4.3	
	Other HD	61	10.1	
	Stroke	18	3.0	
	Varicose veins (legs)	106	17.6	
Musculoskeletal disorders	Arthrosis	205	34.1	43.8
	CBP cervical	200	33.3	
	CBP lumbar	234	38.9	
	Osteoporosis	59	9.8	
Immunological disease	Chronic allergy	209	34.8	34.8
Respiratory disease	Asthma	198	32.9	32.9
Digestive problems	Liver dysfunction	27	4.5	29.8
	Stomach/duodenal ulcer	62	10.3	
	Chronic constipation	58	9.7	
	Haemorrhoids	96	16.0	
Urogenital diseases	Urinary incontinence	66	11.0	25.3
	Kidney problems	56	9.3	
	Prostate problems (men)	34	5.7	
	Menopausal problems (women)	42	13.4	
Eye problems	Cataracts	78	13.0	13.0
Dermatological problems	Chronic skin problems	83	13.8	13.8
Mental health problems	Depression	168	28.0	34.4
	Chronic anxiety	134	22.3	
	Other mental problems	18	3.0	
Neurological disorder	Migraine	145	24.1	
Neoplasias	Malignant tumors	62	10.3	10.3
Endocrinal and metabolic diseases	Thyroid problems	56	9.3	40.8
	Diabetes	99	16.5	
	High cholesterol	184	30.6	
Permanent injuries (accident)		34	11.8	11.8

*n*: Sample size; %: Percentage.

**Table 3:** Association of physical activity (PA) and chronic obstructive pulmonary disease (COPD) comorbidities (outcome) estimated by multivariable logistic regression.

Comorbidities	OR ^1^	CI 95%^1^	*p* ^1^	OR ^2^	CI 95% ^2^	*p* ^2^	OR ^3^	CI 95% ^3^	*p* ^3^
Hypertension	0.935	[0.638–1.370]	0.729	-	-	-	-	-	-
Myocardial infarction	2.171	[0.844–5.586]	0.108	-	-	-	-	-	-
Angina, Coronary HD	0.893	[0.391–2.039]	0.788	-	-	-	-	-	-
Other HD	1.616	[0.949–2.752]	0.077	-	-	-	-	-	-
Stroke	1.801	[0.413–2.831]	0.874	-	-	-	-	-	-
Diabetes	1.594	[1.031–2.463]	0.036 *	1.347	[0.844–2.149]	0.212	-	-	-
High cholesterol	1.060	[0.741–1.516]	0.752	-	-	-	-	-	-
Varicose veins (legs)	0.936	[0.605–1.449]	0.768	-	-	-	-	-	-
Hemorrhoids	1.323	[0.849–2.061]	0.216	-	-	-	-	-	-
Arthrosis	1.548	[1.095–2.187]	0.013 *	1.230	[0.832–1.816]	0.299	-	-	-
CBP cervical	1.365	[0.964–1.933]	0.080	-	-	-	-	-	-
CBP lumbar	1.718	[1.225–2.409]	0.002 *	1.553	[1.09–2.204]	0.014 *	1.489	[1.044–2.125]	0.028 *
Osteoporosis	1.871	[1.090–3.210]	0.023 *	1.713	[0.958–3.064]	0.069	-	-	-
Chronic allergy	0.894	[0.630–1.268]	0.529	-	-	-	-	-	-
Asthma	0.923	[0.648–1.315]	0.658	-	-	-	-	-	-
Liver dysfunction	1.881	[0.867–4.078]	0.110	-	-	-	-	-	-
Stomach/duodenal ulcer	1.6181	[0.991–2.851]	0.054	-	-	-	-	-	-
Chronic constipation	2.268	[1.313–3.918]	0.003 *	2.023	[1.150–3.558]	0.015 *	1.970	[1.119–3.469]	0.019 *
Urinary incontinence	2.568	[1.527–4.317]	0.000 *	2.179	[1.251–3.796]	0.006 *	2.115	[1.213–3.689]	0.008 *
Kidney problems	1.531	[0.880–2.663]	0.132	-	-	-	-	-	-
Cataracts	1.842	[1.141–2.974]	0.012 *	1.918	[1.122–3.279]	0.017*	1.840	[1.074–3.153]	0.026 *
Chronic skin problems	0.897	[0.552–1.457]	0.660	-	-	-	-	-	-
Depression	1.721	[1.197–2.475]	0.003 *	1.494	[1.017–2.194]	0.041 *	1.443	[0.980–2.126]	0.063
Chronic anxiety	1.703	[1.153–2.513]	0.007 *	1.556	[1.036–2.336]	0.033 *	1.508	[1.002–2.269]	0.049 *
Other mental problems	1.724	[0.674–4.410]	0.256	-	-	-	-	-	-
Migraine	1.318	[0.900–1.903]	0.156	-	-	-	-	-	-
Malignant tumors	1.563	[0.921–2.652]	0.098	-	-	-	-	-	-
Thyroid problems	0.858	[0.481–1.533]	0.606	-	-	-	-	-	-
Prostate problems (men)	1.617	[0.782–3.342]	0.195	-	-	-	-	-	-
Menopausal problems (women)	0.818	[0.416–1.609]	0.561	-	-	-	-	-	-
Permanent injuries (accident)	0.807	[0.509–1.278]	0.360	-	-	-	-	-	-

HD: Heart disease; CBP: Chronic back pain; OR: Odd ratio; CI: Confidence interval. * p < 0.05; ^1^ models not adjusted; ^2^ models are adjusted for sex, age, education level, marital status, smoking, alcohol consumption and obesity; ^3^ models are adjusted for sex, age, education level, marital status, smoking, alcohol consumption, obesity, presence of comorbidities and medication intake.
